# Forming of Dynamic Microstructure of Flexible Polymer

**DOI:** 10.3390/ma12203332

**Published:** 2019-10-12

**Authors:** Yung-Jin Weng

**Affiliations:** Department of Mechanical and Energy Engineering, National Chiayi University, Chiayi City 60004, Taiwan; yjweng@mail.ncyu.edu.tw

**Keywords:** bifacial gasbag, microstructure, dynamic forming, cross mold, modeling and simulation

## Abstract

This study focuses on the forming of dynamic microstructure of flexible polymer. The dynamic tensile control microstructure of the polymer mold, along with the gasbag, was used to exert pressure to achieve forming. This study simulated the dynamic control of the flexible mold, and proposed four mechanical models of material viscoelastic response for modeling and evaluation. MATLAB software was used to calculate the imprint prediction calculation theory construction according to the imprint result of curved surface and asymmetric imprint forming. This study designed and developed a gasbag-assisted dynamic forming system, and tested the proposed system for verification. The test results showed that the mechanical stability, curved surface, and asymmetric imprint prediction calculation of the mechanical model of the viscoelastic response of flexible mold material, as proposed in this study, can display the geometric features of the imprinted microstructure. The dynamic mold microstructure control process can accurately transfer a bifacial microstructure and construct the confidence interval for transfer printing forming.

## 1. Introduction

With the rapid advances in science and technology, continuous mass production is the future of industrial development, and system engineering follows the trend of microminiaturization. Under the establishment and development of microsystem technology, microsystems have been rapidly, effectively, and continuously used in science and technology industries. The microelectromechanical system (MEMS) process technology [1,2,3,4] and semiconductor process technology [5,6,7] are critical in the mass production of microsystem components. They are both integrated with multiple technologies, and generally arranged according to multiple sequential processes, including the cleaning and surface preparation of wafer preprocesses, photoresist coating and baking, alignment exposure, development, etching (or ion implantation), etc., as the final process of creating micrographic components or micro molds. The technologies are continuously updated and developed. The modeling process of microsystem components is required before bulk production. In the process, the mold material is usually used for fabricating accurate sized molds, where the MEMS technology is often used, while Mask, Photoresist, and Lithography technologies are used to fabricate microstructure components. As the MEMS technology can achieve forming of stable microstructure components, it has been widely used in the front end of line and middle end of line in the bulk production of most semiconductors. Although the MEMS technology has high accuracy, the equipment cost is high. Moreover, the silicon substrate is the main material, but the hard-brittle (fragile) silicon substrate may increase the defects in the microsystem structure components during bulk production, thus, the fabrication process requires experts in the field. In addition, a precision mold is also time-consuming to fabricate. The model molding technology has developed rapidly in recent years, and the common microstructure component model molding technologies at present include plastics micro-injection molding [8,9], micro-thermoforming [10,11,12], micro-casting [13,14,15], and micro/nano imprinting [16,17,18,19,20,21,22,23,24,25,26,27,28,29,30]. While the aforesaid technologies have their characteristics and merits, most of them require molds for micro-molding processing. In industrial practice, the polymer material, namely polydimethylsiloxane (PDMS), is commonly used for molds. This material is a polymer organic silicon compound with excellent mechanical properties and physical characteristics. Given its excellent molding characteristics during molding, it has been extensively used. In recent years, in order to meet the requirements of academic research and high-tech demands for industrial practices, the optical light guide components or biomedical microstructure components often require microstructures in nonisotropic shapes and multidirectional shapes, or microstructure components with multiple surface shapes. This study proposes a dynamic microstructure forming method for flexible polymer, where the four mold models Maxwell model [31,32,33,34], Kelvin–Voight model [35,36,37,38], Model A, and Model B were built for the cross microstructure mold to find out the steadiest performance of mold materials mechanical properties and surface and asymmetric imprint geometry predictions are dynamically adjusted and established according to the flexibility of PDMS in the elastic range. It further proposes a forming operation window and confidence interval of transfer printing forming for the developed system. System stability is tested by actual experiments to discuss this innovative dynamic microstructure forming process for flexible polymer. 

## 2. Construction and Analysis of the Theoretical Mechanism of Dynamic Tensile Deformation 

### 2.1. Building and Evaluation of Mechanical Model of Viscoelastic Response of Mold Material 

This study used the axial tensile method as the basis of flexible mold microstructure shape control; therefore, under the fixed stress, the creep phenomenon may occur with the tensile time. This study used Maxwell, Kelvin–Voight, ModelA, and ModelB for modeling and discussion. 

#### 2.1.1. Maxwell Model and Kelvin–Voight Model Building 

**Figure 1:**
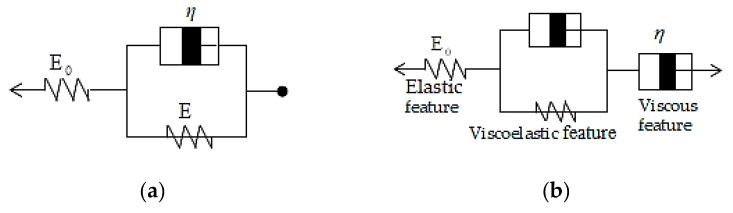
Flexible polymer mold viscoelastic response (**a**) Maxwell model (**b**) creep prediction model.

This study set the Maxwell and Kelvin–Voight models as the first series of construction and evaluation method for mold dynamic tensile mechanical properties [39], as shown in Figure 1a, in order to determine the exact geometric deformation size and variation of the cross microstructure mold of this study in the dynamic tensile process. Regarding the mold material mechanical properties for model building, the damper and spring were used as the basic modules for mechanical model building. The total stress is expressed as Equation (1). 

Maxwell model:

(1) σ=E0εe+η(dε0dt)

Given the fixed tensile, the estimated tensile stress (σ) is fixed, and then

dεdt+(Eη)ε=1η(E0+EE0)σ0

Let, (k=ddt, j=Eη, m, C be constants) 

After mathematical analysis, as expressed by Equation (2)

(2) ε=mj+Ce-jt=(E0+EE0E)σ0+Ce-jt

According to the elastic strain in the initial stress range, ε(t=0)=ε0=σ0E0 and then

σ0E0=(E0+EE)σ0E0+C

where, C=−σ0E;

Thus, for time (t), the strain changes are shown by Equation (3)

(3) ε=(E0+EE0E)σ0−(σ0E)e−jt=σ0E0+σ0E(1−e−jt)

After elastic strain, as the strain increases, the velocity gradually reduces, reaching the final strain ($\varepsilon_{final}$) state, as expressed by Equation (4)

(4) εfinal=σ0E0+σ0E

Based on the above equations, the creep deformation mode can be expressed by Equation (5) for prediction computation, as shown in Figure 1b.

(5) ε=σ0E0+σ0E(1−e−jt)+(σ0η0)t

In addition, as the tensile is maintained in the dynamic tensile process, the polymer may have stress relaxation in this process, which is the key point of evaluation. This study sets the tensile deformation as a constant ($\varepsilon_{0}$), the Maxwell model is used as the evaluation method. The three-parameter model is used to discuss and spread the preliminary equations, as expressed by Equation (6)

(6) dεdt+(Eη)ε=1η(E0+EE0)σ0

After further compilation:

dσdt+(E0+EE0)σ0=(Eη)σ0

However, due to $\sigma_{0}=E_{0}\varepsilon_{0}$, after a long time, the final stress relaxation value is expressed as Equation (7)

(7) σfinal=Eσ0E0+E

In this study, the dynamic tensile is to control microstructure deformation, and this action is repeated and compared with stress relaxation, thus, the polymer creep phenomenon is more important. 

The significance of the polymer is defined, as follows: 

where, S: Total tensile stress; $S_{e}$: Tensile stress on elastic modulus; $S_{a}$: Tensile stress on viscous modulus; 

ε: Total tensile strain; $\varepsilon_{e}$: Tensile strain on elastic modulus; $\varepsilon_{a}$: Tensile strain on viscous modulus 

**Figure 2:**
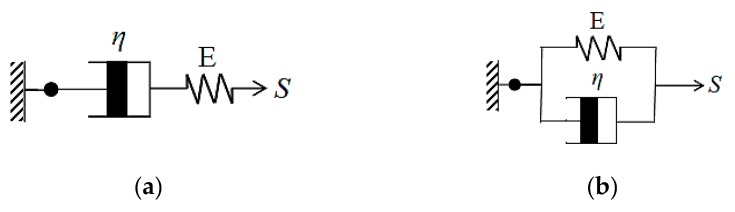
Creep prediction models (**a**) Maxwell model (**b**) Kelvin–Voight model.

Therefore, after the Maxwell model is compiled (Figure 2a), it is expressed as Equation (8)

(8) $$\frac{d\varepsilon }{dt}=\frac{1}{E}\frac{dS_{e}}{dt}+\frac{S_{a}}{\eta }$$

In a similar manner, the Kelvin–Voight model can be deduced (Figure 2b), and expressed as Equation (9)

(9) $$S=E\varepsilon +\eta \frac{d\varepsilon }{dt}$$

#### 2.1.2. Constructing Model A and Model B 

**Figure 3:**
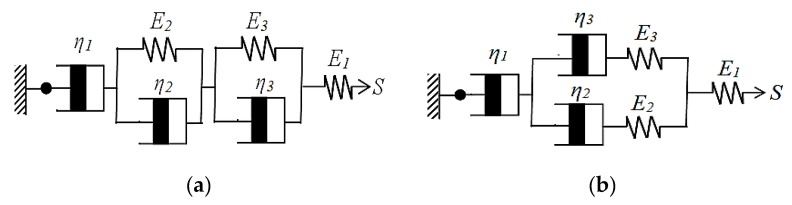
Creep prediction models (**a**) Model A (**b**) Model B.

The second series Model A and the complex in-tandem models are built, as shown in Figure 3a. The complex in-tandem model, Model B, is built, as shown in Figure 3b. The mechanical properties of mold dynamic tensile are established and evaluated, in order to determine the optimum model. 

Model A: 

$\eta_{1}$: the first viscous modulus; $\eta_{2}$: the second viscous modulus; η3 : the third viscous modulus 

$E_{1}$: the first elastic modulus; $E_{2}$: the second elastic modulus; $E_{3}$: the third elastic modulus; S: Tensile stress

(10) $$S=S_{e}=S_{2}=S_{3}=S_{a}$$

where, $S_{e}$: Tensile stress on the first elastic modulus; $S_{2}$: Tensile stress on lower Kelvin–Voight 

$S_{3}$: Tensile stress on upper Kelvin–Voight; $S_{a}$: Tensile stress on the first viscous modulus;

(11) $$\varepsilon =\varepsilon_{e}+\varepsilon_{2}+\varepsilon_{3}+\varepsilon_{a}$$

where, ε: Total tensile strain; $\varepsilon_{e}$: Tensile strain on the first elastic modulus; $\varepsilon_{2}$: Tensile strain on lower Kelvin–Voight; $\varepsilon_{3}$: Tensile strain on upper Kelvin–Voight; $\varepsilon_{a}$: Tensile strain on the first viscous modulus;

(12) $$\frac{d\varepsilon }{dt}=\frac{d\varepsilon_{e}}{dt}+\frac{d\varepsilon_{2}}{dt}+\frac{d\varepsilon_{3}}{dt}+\frac{d\varepsilon_{a}}{dt}=\frac{1}{E_{1}}\frac{dS_{e}}{dt}+\frac{S_{2}-E_{2}\varepsilon_{2}}{\eta_{2}}+\frac{S_{3}-E_{3}\varepsilon_{3}}{\eta_{3}}+\frac{S_{a}}{\eta_{1}}$$

$\varepsilon_{e}$: can be replaced by $\frac{S_{e}}{E_{1}};\;\frac{d\varepsilon_{2}}{dt}$: can be replaced by $\frac{S_{2}-E_{2}\varepsilon_{2}}{\eta_{2}};\;\frac{d\varepsilon_{3}}{dt}$: can be replaced by $\frac{S_{3}-E_{3}\varepsilon_{3}}{\eta_{3}};\;\frac{d\varepsilon_{a}}{dt}$: can be replaced by $\frac{S_{a}}{\eta_{1}}$

$$\frac{d\varepsilon }{dt}+\frac{E_{2}\varepsilon_{2}}{\eta_{2}}+\frac{E_{3}\varepsilon_{3}}{\eta_{3}}=\frac{1}{E_{1}}\frac{dS_{e}}{dt}+\frac{S_{2}}{\eta_{2}}+\frac{S_{3}}{\eta_{3}}+\frac{S_{a}}{\eta_{1}}$$

Reduced to Equation (13):

(13) $$\frac{d\varepsilon }{dt}+\frac{E_{2}\varepsilon_{2}}{\eta_{2}}+\frac{E_{3}\varepsilon_{3}}{\eta_{3}}=\frac{1}{E_{1}}\frac{dS}{dt}+\frac{S}{\eta_{2}}+\frac{S}{\eta_{3}}+\frac{S}{\eta_{1}}$$

Let,$\;E_{1},{\;E}_{2},{\;E}_{3}$ be replaced by $E;{\;\eta }_{1},{\;\eta }_{2},{\;\eta }_{3}$ can be replaced by η, then

$$\frac{d\varepsilon }{dt}+\frac{E\varepsilon_{2}}{\eta }+\frac{E\varepsilon_{3}}{\eta }=\frac{1}{E}\frac{dS}{dt}+\frac{S}{\eta }+\frac{S}{\eta }+\frac{S}{\eta }$$

Let,$\;\varepsilon_{2}+\varepsilon_{3}$ be replaced by $\frac{\varepsilon }{2}$, then, the Model A mold creep equation can be obtained, and expressed as Equation (14)

(14) $$\frac{d\varepsilon }{dt}+\frac{E}{\eta }\frac{\varepsilon }{2}=\frac{1}{E}\frac{dS}{dt}+3\frac{S}{\eta }$$

Model B: 

$\eta_{1}$: the first viscous modulus; $\eta_{2}$: the second viscous modulus; $\eta_{3}$: the third viscous modulus 

$E_{1}$: the first elastic modulus; $E_{2}$: the second elastic modulus; $E_{3}$: the third elastic modulus; S: Tensile stress

(15) $$S=S_{e}=S_{23}=S_{a}$$

where, $S_{e}$: Tensile stress on the first elastic modulus; $S_{23}$: Total tensile stress at parallel joint; $S_{a}$: Tensile stress on the first viscous modulus;

(16) $$\varepsilon =\varepsilon_{e}+\varepsilon_{23}+\varepsilon_{a}$$

where, ε: Tensile strain; $\varepsilon_{e}$: Tensile strain on the first elastic modulus; $\varepsilon_{23}$: Total tensile strain at parallel joint; $\varepsilon_{a}$: Tensile strain on the first viscous modulus

(17) $$\frac{d\varepsilon }{\mathrm{dt}}=\frac{d\varepsilon_{e}}{dt}+\frac{d\varepsilon_{23}}{\mathrm{dt}}+\frac{d\varepsilon_{a}}{dt}=\frac{1}{E_{1}}\frac{dS_{e}}{dt}+\frac{\left(S_{23}+\left(\frac{\eta_{2}}{2E_{2}}+\frac{\eta_{3}}{2E_{3}}\right)\frac{{\mathrm{dS}}_{23}}{\mathrm{dt}}\right)}{\left(\eta_{2}+\eta_{3}\right)}+\frac{S_{a}}{\eta_{1}}$$

Let,$\;\varepsilon_{e}$ be replaced by $\frac{S_{e}}{E_{1}}$

(18) $$\varepsilon_{23}=\varepsilon_{2}=\varepsilon_{3}\;;\;\varepsilon_{2}=\varepsilon_{2e}+\varepsilon_{2a}\;;\;\varepsilon_{3}=\varepsilon_{3e}+\varepsilon_{3a}$$

(19) $$S_{23}=S_{2}+S_{3}$$

where, $\varepsilon_{2}$: Tensile strain on the left of parallel joint; $\varepsilon_{3}$: Tensile strain on the right of parallel joint; $S_{2}$: Tensile stress on the left of parallel joint; $S_{3}$: Tensile stress on the right of parallel joint

(20) $$S_{2}=S_{2e}=S_{2a}=\eta_{2}\frac{d\varepsilon_{2a}}{dt}=\eta_{2}\frac{d\left(\varepsilon_{2}-\varepsilon_{2e}\right)}{\mathrm{dt}}=\eta_{2}\frac{{d\varepsilon }_{2}}{\mathrm{dt}}-\eta_{2}\frac{{d\varepsilon }_{2e}}{\mathrm{dt}}$$

where, $S_{2e}$: Tensile stress on the second elastic modulus on the left of parallel joint; $S_{2a}$: Tensile stress on the second viscous modulus on the left of parallel joint, and can be replaced by $\;\;\eta_{2}\frac{d\varepsilon_{2}}{dt}$

(21) $$S_{3}=S_{3e}=S_{3a}=\eta_{3}\frac{d\varepsilon_{3a}}{dt}=\eta_{3}\frac{d\left(\varepsilon_{3}-\varepsilon_{3e}\right)}{\mathrm{dt}}$$

where, $S_{3e}$: Tensile stress on the third elastic modulus on the right of parallel joint; $S_{3a}$: Tensile stress on the third viscous modulus on the right of parallel joint, and can be replaced by $\eta_{3}\frac{d\varepsilon_{3}}{dt}$

(22) $$S_{23}=\eta_{2}\frac{d\varepsilon_{2}}{dt}-\eta_{2}\frac{{d\varepsilon }_{2e}}{\mathrm{dt}}+\eta_{3}\frac{d\varepsilon_{3}}{dt}-\eta_{3}\frac{{d\varepsilon }_{3e}}{\mathrm{dt}}$$

(23) $$S_{23}=\eta_{2}\frac{d\varepsilon_{23}}{dt}-\eta_{2}\frac{{d\varepsilon }_{2e}}{\mathrm{dt}}+\eta_{3}\frac{d\varepsilon_{23}}{dt}-\eta_{3}\frac{{d\varepsilon }_{3e}}{\mathrm{dt}}=\eta_{2}\frac{{d\varepsilon }_{23}}{\mathrm{dt}}-\frac{\eta_{2}}{E_{2}}\frac{{\mathrm{dS}}_{2}}{\mathrm{dt}}+\eta_{3}\frac{{d\varepsilon }_{23}}{\mathrm{dt}}-\frac{\eta_{3}}{E_{3}}\frac{{\mathrm{dS}}_{3}}{\mathrm{dt}}$$

Let, $\varepsilon_{2e}$ be replaced by $\frac{S_{2}}{E_{2}}$; $\varepsilon_{3e}$ be replaced by $\frac{S_{3}}{E_{3}}$

(24) $$S_{2}=S_{3}=\frac{S_{23}}{2}=\frac{\left(\eta_{2}\frac{{d\varepsilon }_{23}}{\mathrm{dt}}-\frac{\eta_{2}}{2E_{2}}\frac{{\mathrm{dS}}_{23}}{\mathrm{dt}}+\eta_{3}\frac{{d\varepsilon }_{23}}{\mathrm{dt}}-\frac{\eta_{3}}{2E_{3}}\frac{{\mathrm{dS}}_{23}}{\mathrm{dt}}\right)}{2}\;\frac{d\varepsilon_{23}}{\mathrm{dt}}=\frac{\left(S_{23}+\left(\frac{\eta_{2}}{2E_{2}}+\frac{\eta_{3}}{2E_{3}}\right)\frac{{\mathrm{dS}}_{23}}{\mathrm{dt}}\right)}{\left(\eta_{2}+\eta_{3}\right)}$$

Let, $\frac{d\varepsilon_{a}}{dt}$ be replaced by $\frac{S_{a}}{\eta_{1}}$;

(25) $$\frac{d\varepsilon }{dt}=\frac{1}{E_{1}}\frac{dS}{dt}+\frac{\left(S+\left(\frac{\eta_{2}}{2E_{2}}+\frac{\eta_{3}}{2E_{3}}\right)\frac{\mathrm{dS}}{\mathrm{dt}}\right)}{\left(\eta_{2}+\eta_{3}\right)}+\frac{S}{\eta_{1}}$$

Let,$\;E_{1}$ be replaced by E; $\eta_{1},{\;\eta }_{2},{\;\eta }_{3}$ be replaced by η

(26) $$\frac{d\varepsilon }{\mathrm{dt}}=\frac{1}{E}\frac{dS}{dt}+\frac{\left(S+\left(\frac{\eta }{2E}+\frac{\eta }{2E}\right)\frac{\mathrm{dS}}{\mathrm{dt}}\right)}{\left(\eta +\eta \right)}+\frac{S}{\eta }=\frac{1}{E}\frac{\mathrm{dS}}{\mathrm{dt}}+\frac{1}{2E}\frac{\mathrm{dS}}{\mathrm{dt}}+\frac{1}{2}\frac{S}{\eta }+\frac{S}{\eta }$$

Then, the Model B mold creep equation can be obtained, and expressed as Equation (27)

(27) $$\frac{d\varepsilon }{\mathrm{dt}}=\frac{3}{2}\left(\frac{1}{E}\frac{\mathrm{dS}}{\mathrm{dt}}+\frac{S}{\eta }\right)$$

#### 2.1.3. Asymmetric Imprint Molding Prediction Geometric Shape Construction 

**Figure 4:**
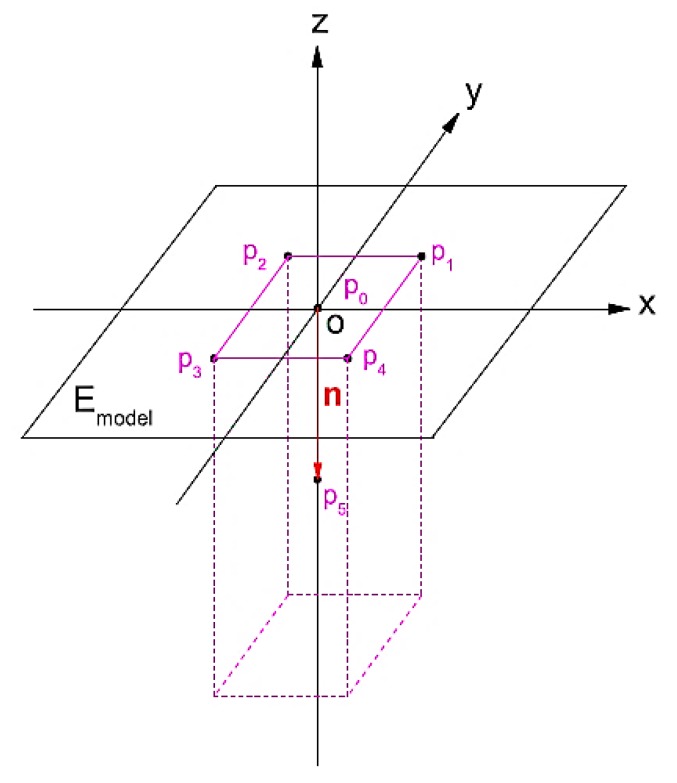
Rectangular microstructure calculation theory construction pattern.

This study used the array rectangular microstructure to establish the computational theory of the asymmetric imprint molding prediction method. The square column arrays microstructure is geometrically calculated. MATLAB software is used to establish the asymmetric imprint prediction calculation theory. Considering the square column arrays, the appearance of the nanoimprint patterns of each square column exhibits a rhomb shape. Referring to Weng’s study [40] of the asymmetric theorem, this study set a single rectangular microstructure (Figure 4), array rectangular microstructure, array cylindrical microstructure, and the array microlens hemisphere structure for asymmetric imprint calculation prediction using MATLAB.

## 3. Experimental

### 3.1. Design and Development of the Gasbag-Assisted Dynamic Forming System with Microstructure Mold Preparation 

**Figure 5:**
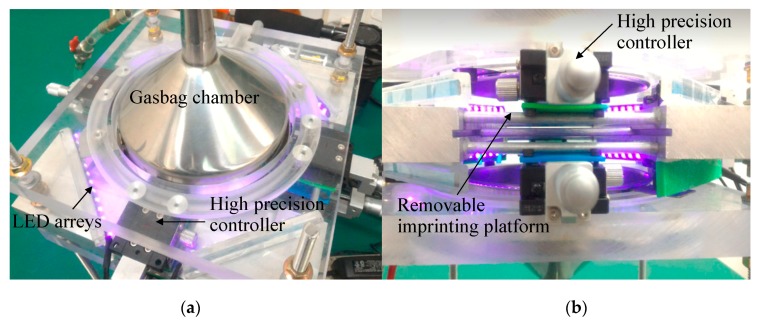
Innovative gasbag-assisted dynamic forming system. (**a**) Top view (**b**) Side view.

This study developed an innovative gasbag-assisted dynamic forming system, which can perform single-sided and simultaneous bifacial gasbag-assisted dynamic forming. The forming resists are exposed and cured by a side array UV-LED lamp, as shown in Figure 5. The imprinting system developed in this study is comprised of the following main components: upper and lower filling gasbag cavities (filling pressure gas), a changeable transparent imprinting platform in the middle, eight array UV exposure systems on the side, and 8 angle adjustable high accuracy microstructure mold dynamic fine regulators in the middle. The proposed system can perform single-sided operations or unilateral exposure, and is flexible in the forming process. The mold microstructure is made by the laser processing of aluminum sheets and casting, and the cross microstructure mold material is PDMS (Polydimethylsiloxane, Sylgard™ 184, Dow Corning). 

### 3.2. Gasbag-Assisted Compressive Stress Distribution Simulation and Microstructure Change Analysis 

This study used Abaqus and ANSYS simulation software to analyze the gasbag-assisted pressure on cross mold stress and strain uniformity, in order to analyze the uniformity of gasbag filling pressure. The uniformity of stress on the cross microstructure mold in the tensile and forming processes was also analyzed to compare the errors in the actual experiment. In addition, the MATLAB software was used to predict the curvature change of the microstructure under pressure in the curved imprinting process for the preliminary identification of the curved imprint. The MATLAB software also calculated the microstructure replication forming shape under the stress of the asymmetric imprinting platform. The results were compared with the actual forming to verify the feasibility and effectiveness of the proposed system and prediction method. 

### 3.3. Gasbag-Assisted Dynamic Microstructure Forming Uniformity Test and Molding Steps 

The proposed gasbag-assisted dynamic forming system regulates unequal gas gage pressure. The imprint uniformity was tested by pressure sensitive adhesive tape. The experimental results showed the uniformly distributed pressure color blocks. The system forming steps include: (a) the cross microstructure mold is set up and put in the imprinting plate; (b) the location of the cross microstructure mold is regulated dynamically and the gasbag is filled to generate the force of impression, which is uniformly applied to the mold; (c) the relative imprinted depth of the imprinting plate and cross microstructure mold is controlled and the LED lamp is turned on for exposure forming; (d) the LED lamp is turned off and the gasbag is decompressed to obtain the finished microstructure component after transfer printing. 

## 4. Results and Discussion

### 4.1. Discussion of the Mechanical Simulation of Cross Mold Dynamic Control and Mechanical Model Building 

#### 4.1.1. Cross Mold Dynamic Control Simulation Analysis 

**Figure 6:**
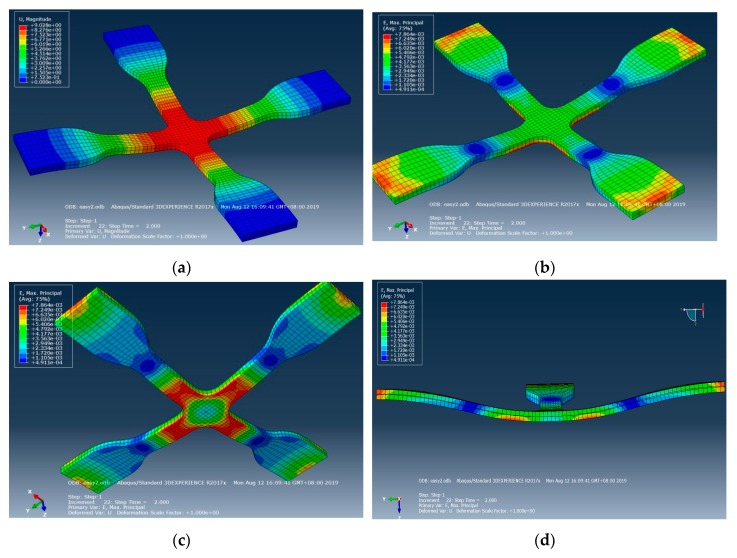
Cross mold contact stress surface, microstructure surface displacement, strain, and cross-section simulation. (**a**) Displacement (stress surface), (**b**) strain (stress surface), (**c**) strain (microstructures surface), (**d**) cross section.

**Figure 7:**
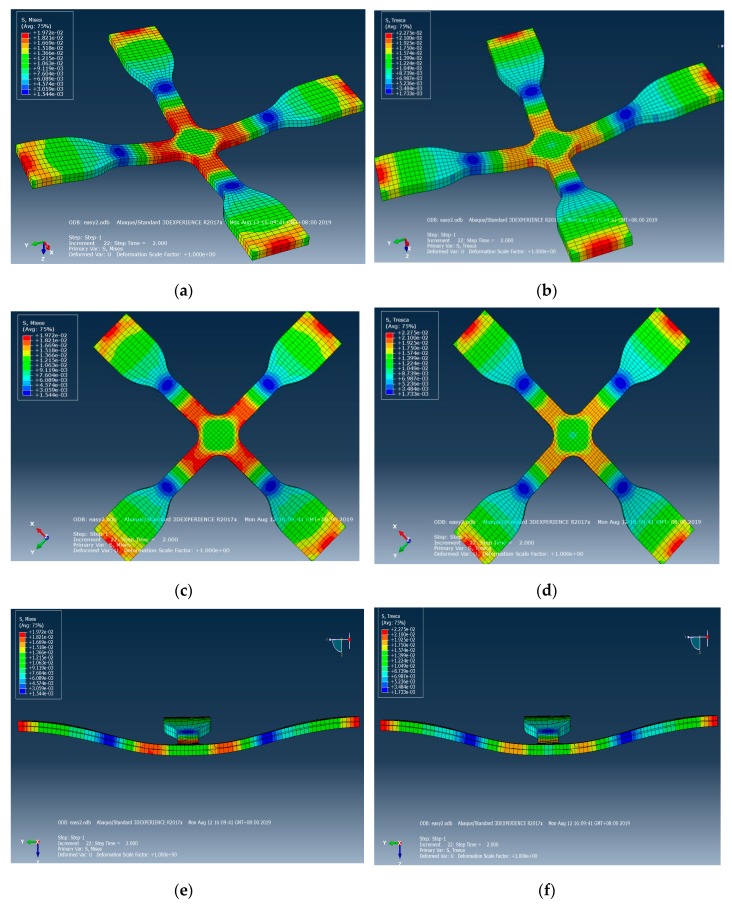
Cross-mold contact stress surface, microstructure surface, cross-section von Mises stress, and Tresca stress distribution simulation. (**a**) Stress surface (von Mises stress) (**b**) Stress surface (Tresca stress) (**c**) Microstructures surface (von Mises stress) (**d**) Microstructures surface (Tresca stress) (**e**) Cross section (von Mises stress) (**f**) Cross section (Tresca stress).

**Figure 8:**
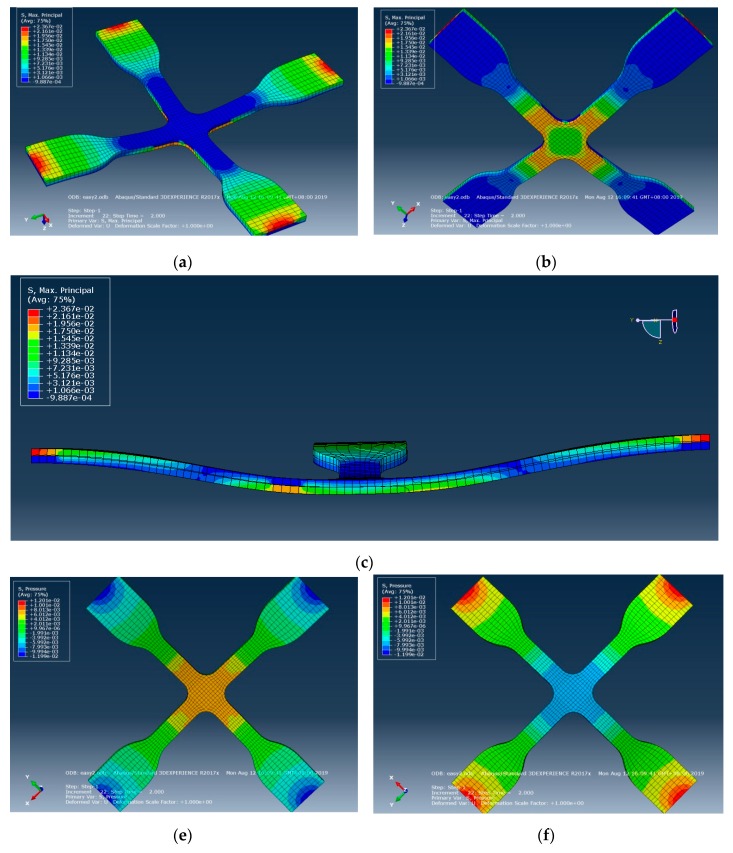
Simulation of force distribution before and after gasbag pressure on cross mold contact stress surface, microstructure surface, and cross section controlled by dynamic tensile. (**a**) Elongation state (Stress surface); (**b**) elongation state (microstructures surface); (**c**) elongation state (cross section); (**d**,**e**) elongation state with gasbag pressure (stress surface); (**f**) elongation state with gasbag pressure (microstructures surface).

This study used Abaqus software for the simulation analysis of the related factors of cross mold in the actual process. After the mechanical properties of material were tested, various material parameters of the system were established as simulation parameters, as shown in Table 1. The simulation results showed that after the gasbag is uniformly filled with inert gas, the stress surface contacts the cross mold, and the microstructure surface and cross section have uniform displacement and strain, as shown in Figure 6. By von Mises stress and Tresca stress, the cross mold is in contact with the stress surface, microstructure surface, and cross section, displaying uniform and symmetric stress distribution, as shown in Figure 7. After dynamic tensile control, before and after the cross mold receives the gasbag pressure, the stress surface, microstructure surface, and cross section have highly uniform force distribution, as shown in Figure 8. As indicated above, the cross sections in all directions of the cross mold have highly uniform and symmetrical stress and strain. 

#### 4.1.2. Construction of Mold Mechanical Model 

**Figure 9:**
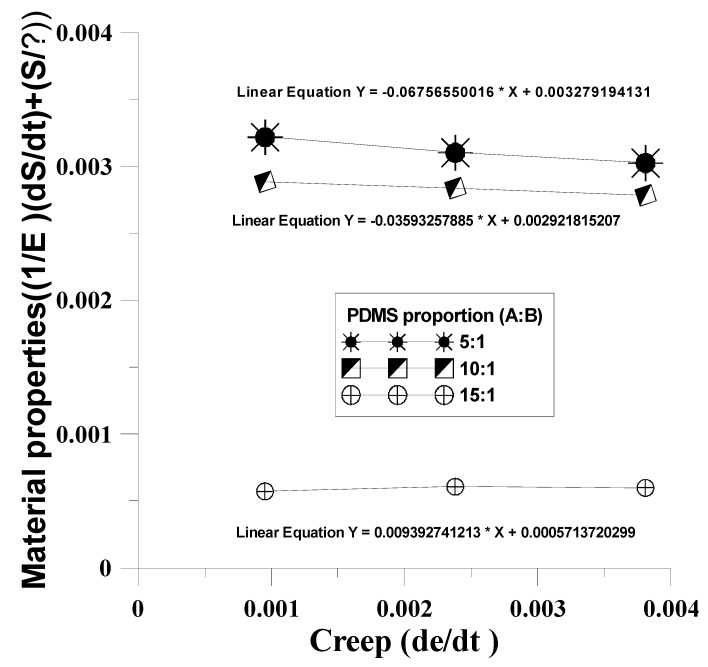
Time-dependent creep behavior of dependent variables in the dynamic tensile process of the Maxwell model.

**Figure 10:**
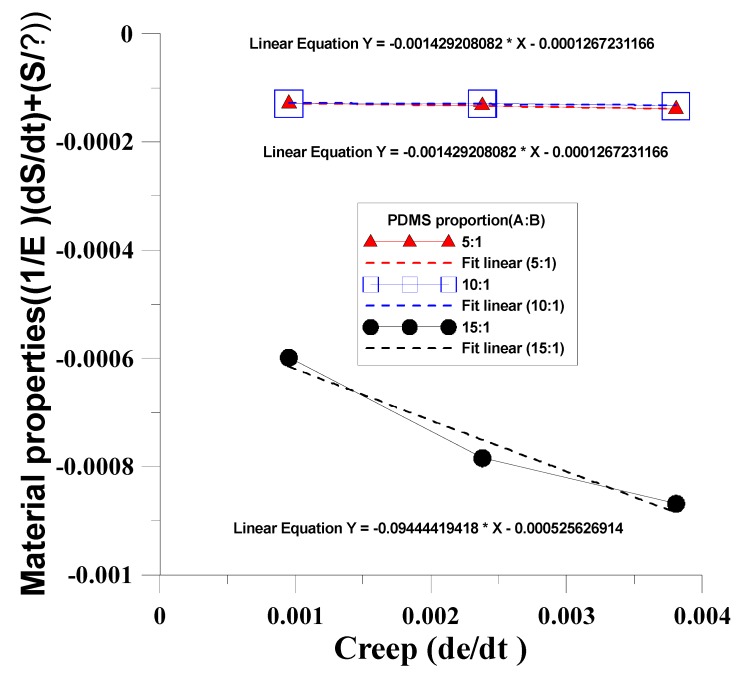
Time-dependent creep behavior of the dependent variables in the dynamic tensile process of the Kelvin–Voight model.

**Figure 11:**
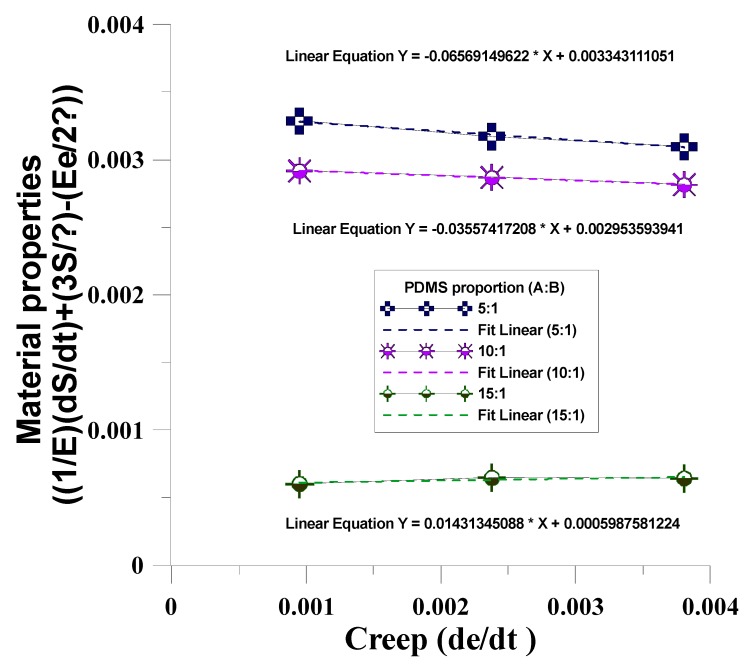
Time-dependent creep behavior of the dependent variables in the dynamic tensile g process of Model A.

**Figure 12:**
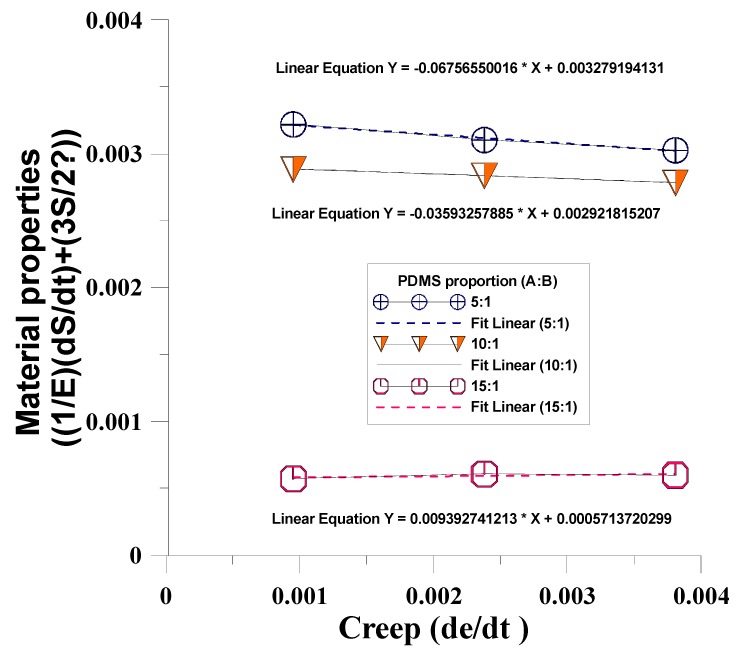
Time-dependent creep behavior of the dependent variables in the dynamic tensile process of Model B.

This study used different mixing ratios of Agent A and Agent B (5:1, 10:1, 15:1) for the mold material (PDMS) to discuss the creep phenomenon after dynamic regulation of the four mechanical models. The mechanical properties test data of PDMS material under unequal test conditions (Table 2) were substituted in the four mechanical models to obtain the η value in the mechanical model (Table 3). The results were compared with the η value of the four mechanical model creep equations to obtain the time-dependent creep behavior of dependent variables in the dynamic tensile process, as shown in Figure 9, Figure 10, Figure 11 and Figure 12. After the four models were built according to the different mixing ratios of Agent A and Agent B, and according to the actual test, the Kelvin–Voight model cannot effectively correspond when the mixing ratio is 15:1 (less able to display the mold materials mechanical properties, as shown in Figure 9), while the precisions of the other three mold mechanical models of sequence are Model A, Model B, and the Maxwell model, that the material properties with respect to creep are more stable, as shown in Figure 10, Figure 11 and Figure 12. This study used Model A as the model to consider creep in the experimental process. 

### 4.2. Curved Imprinting Simulation and Experimental Analysis 

#### 4.2.1. Effect of Curved Imprinting on Mold Microstructure 

**Figure 13:**
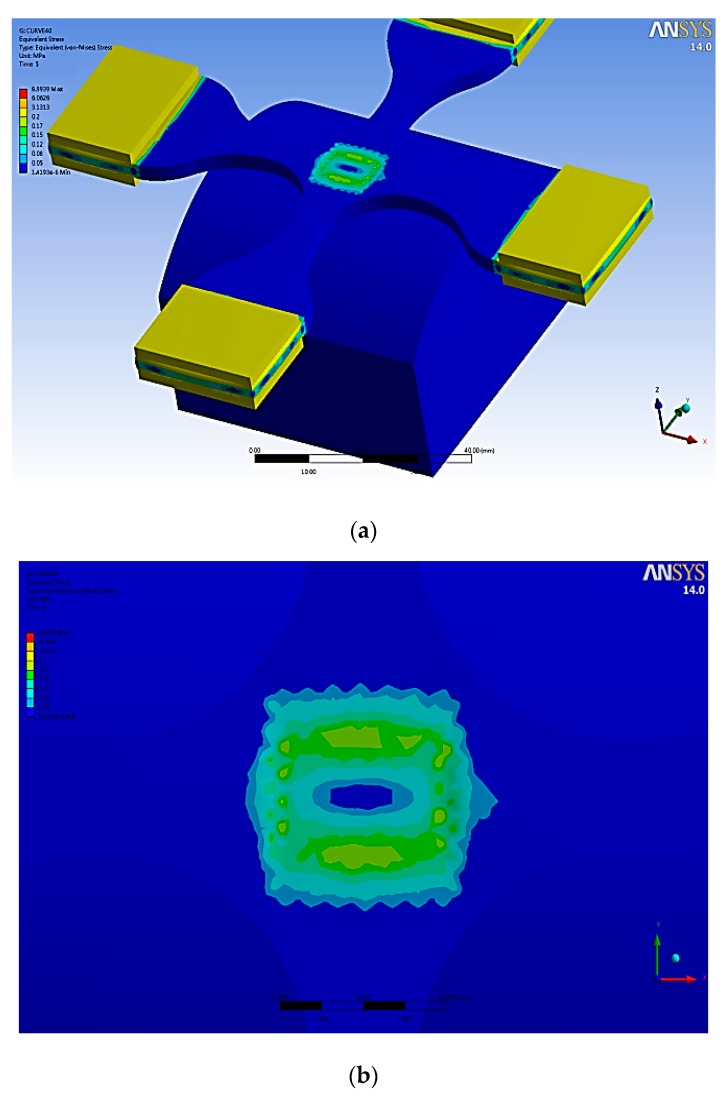
Cross microstructure mold von Mises stress distribution. (**a**) Full view; (**b**) partial view.

This study designed the replaceable imprinting platform as the curved surface (convex, concave) to discuss the changes in the mold microstructure in the imprinting process of the convex surface of the imprinting platform, where the material parameters are given (modulus of elasticity: 1.70 Mpa, Poisson’s ratio: 0.495, density: 965 kg/m^3^). ANSYS software was used to perform the simulation, under imprinting pressure of 0.12 Mpa and surface fillet of R42mm without tensile. According to von Mises stress simulation distribution, the central region of the surface was subject to higher stress, as shown in Figure 13. 

#### 4.2.2. Effect of Curved Imprinting Under Unequal Curvature Radius on Mold Microstructure 

**Figure 14:**
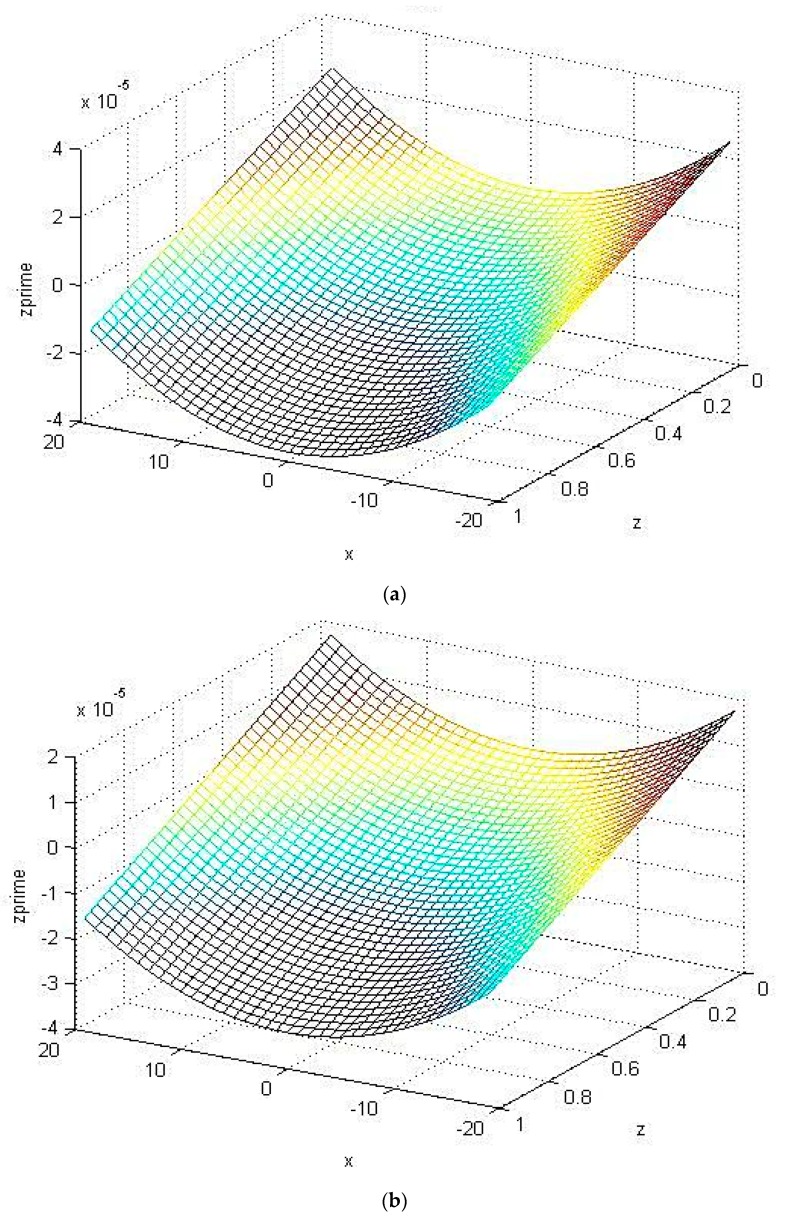
Longitudinal deformation of the microstructure with unequal surface curvature radius. (**a**) Comprehensive deformation (Radius: 25 mm); (**b**) comprehensive deformation (Radius: 30 mm).

**Figure 15:**
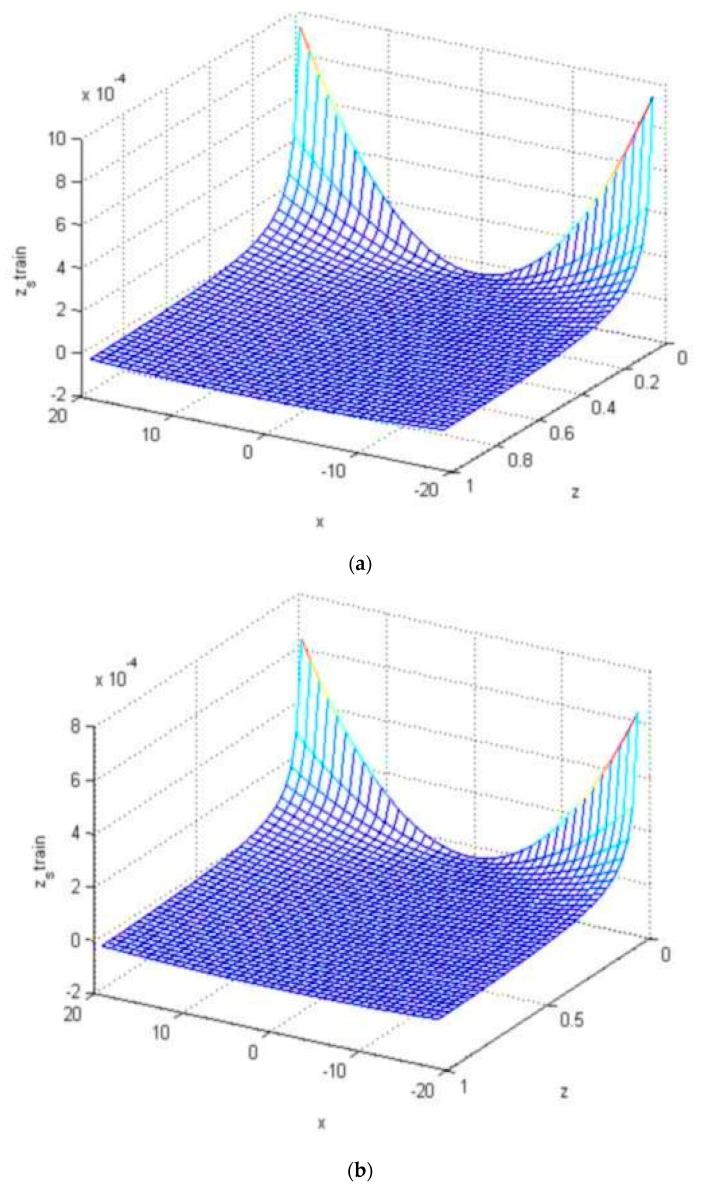
Longitudinal strain of the microstructure with unequal surface curvature radius. (**a**) Comprehensive strain (radius: 25 mm); (**b**) comprehensive strain (radius: 30 mm).

When the imprinting platform was designed as a concave surface, MATLAB software was used to analyze the changes in the cross mold microstructure under unequal concave curvature radius surface (25 mm, 30 mm). This study used a columnar microstructure for analysis. According to the analysis result, with a small curvature radius (25 mm), the longitudinal deformation and strain of the microstructure of the curved surface are larger than those of the curved surface with a large curvature radius (30 mm); the lower the microstructure, the lower the central area of the mold, the larger the longitudinal deformation and strain, and the larger the longitudinal deformation and strain on the mold edge, as shown in Figure 14 and Figure 15, respectively. 

#### 4.2.3. Curved Imprinting Dynamic Regulation Microstructure Replication Forming Analysis 

**Figure 16:**
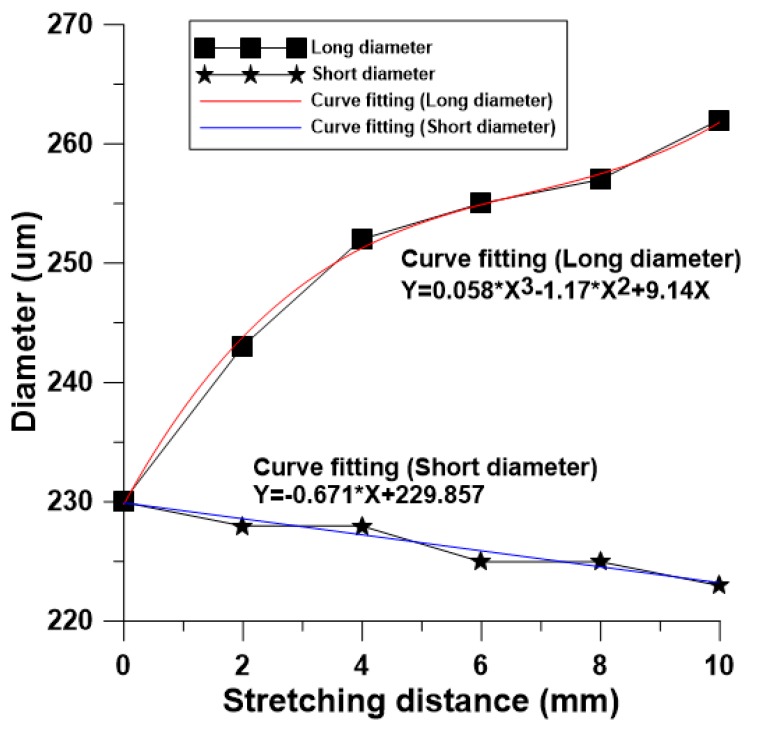
Influence of surface imprinting dynamically controlled uniaxial tensile on microstructure form diameter.

**Figure 17:**
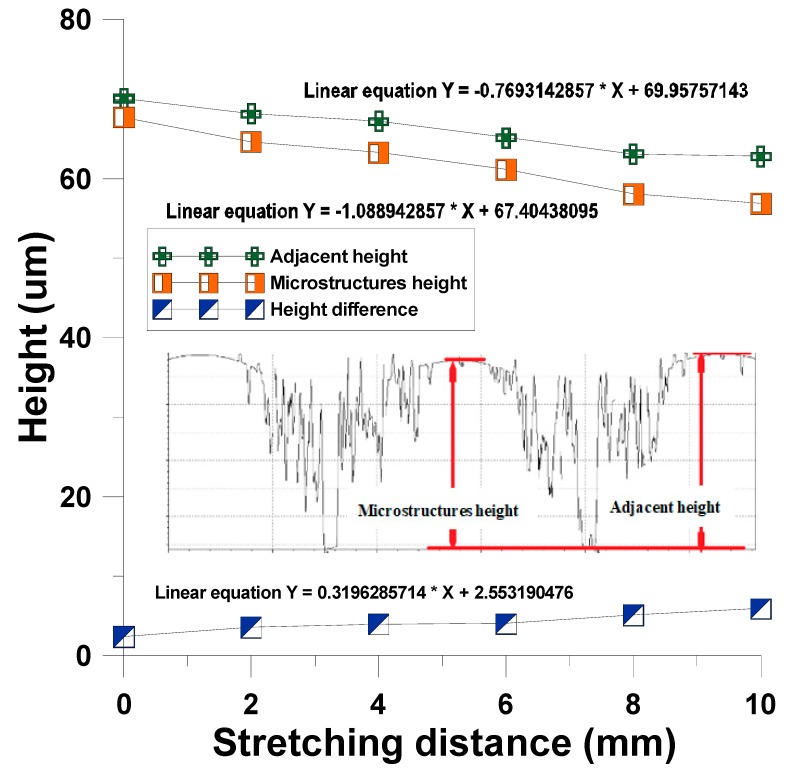
Influence of surface imprinting dynamically controlled uniaxial tensile on microstructure forming height.

This study built an array cylinder with a diameter of 250 μm and a height of 75 μm in a cross microstructure mold. In the actual curved imprinting process, the uniaxial extension of the cross mold microstructure was adjusted (0, 2, 4, 6, 8, 10 mm), pressurized to 0.15 Mpa, and the pressure was maintained. After UV exposure, it was measured by a white light microscope, and the height of the microstructure adjacent to the lowest columnar microstructure was close to the simulation analysis, as shown in Table 4, Figure 16 and Figure 17. 

### 4.3. Asymmetric Imprint MATLAB Prediction Analysis and Bifacial Gasbag Dynamic Experiment Result 

#### 4.3.1. Asymmetric Imprint MATLAB Prediction Analysis and Experiment Accuracy 

**Figure 18:**
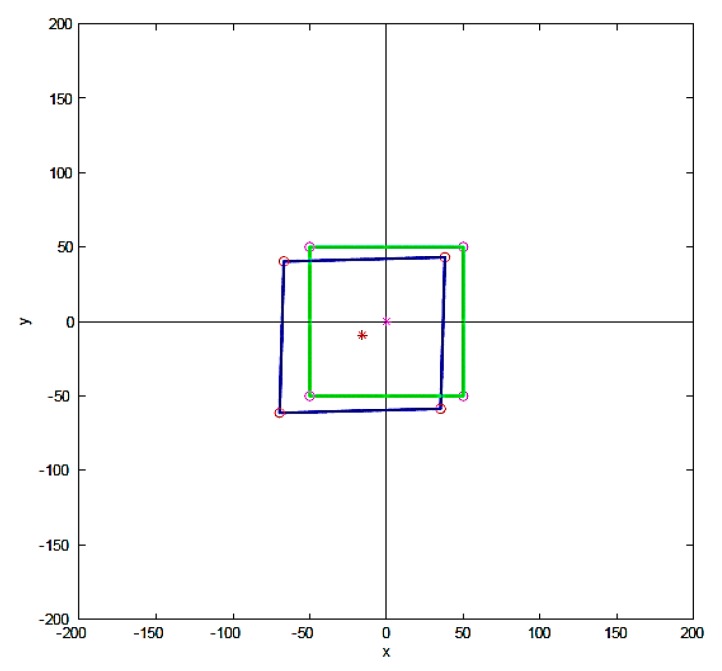
MATLAB calculation predicts single rectangular microstructure rotation angle π/9°, tilt angle π/6°.

**Figure 19:**
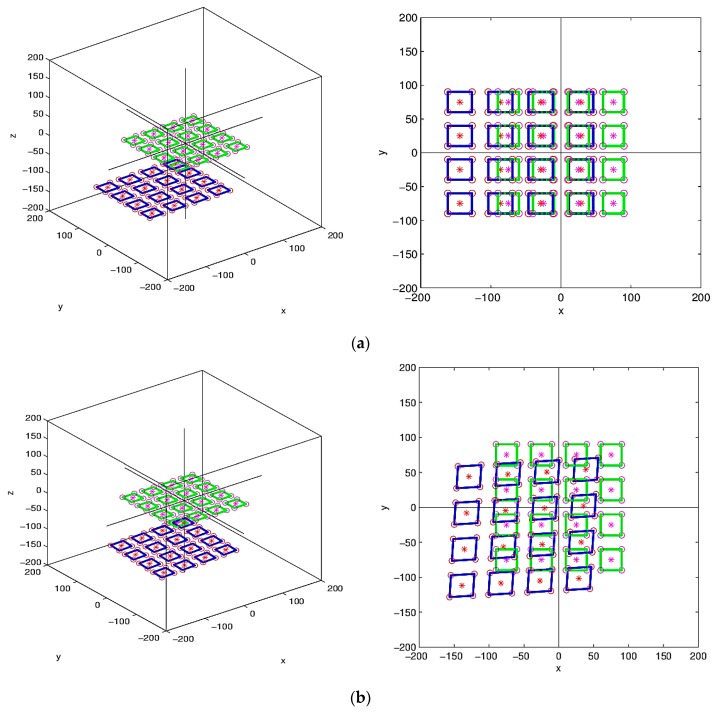
MATLAB calculation predicts array rectangular microstructure (4 × 4). (**a**) Rotation angle 0°, tilt angle π/6°; (**b**) rotation angle π/6°, tilt angle π/6°.

**Figure 20:**
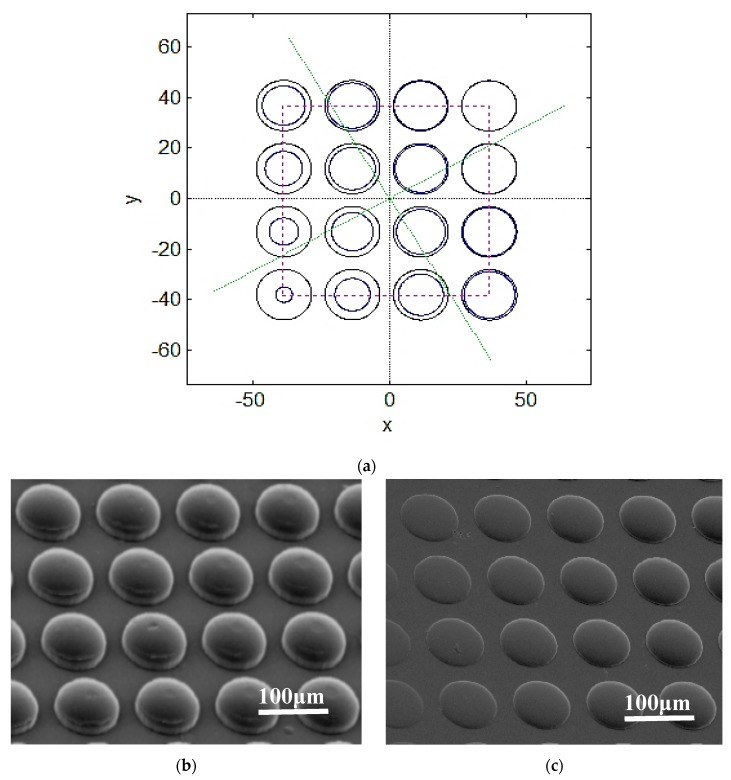
Array microlens hemispherical structure. (**a**) Rotation angle π/6°, tilt angle π/36° MATLAB calculation prediction result; (**b**) rotation angle 0°, tilt angle 0°, actual imprint reproduction result; (**c**) rotation angle π/6°, tilt angle π/36° actual imprint reproduction result.

This study built a rectangular microstructure for prediction analysis, and the imprint forecast result of the unequal angle was calculated. The predicted position variation was calculated according to a single rectangular microstructure (1 × 1) at rotation angle π/9° and tilt angle π/6°, as shown in Figure 18. The calculation prediction of the array rectangular microstructure (4 × 4), where the results of array (4 × 4) at rotation angle 0°, tilt angle π/6° and rotation angle π/ 6°, tilt angle π/6°, is shown in Figure 19. The dynamic tensile asymmetric imprinting experiment was performed. The array microlens hemispherical structure, as derived from the actual dynamic tensile, was reproduced by rotation angle π/6° and tilt angle π/36°. This result is close to the calculation result of MATLAB software, which is enough to show the imprinting stability of this system and the feasibility of the prediction method, as shown in Figure 20. 

#### 4.3.2. Experimental Performance of Bifacial Gasbag-Assisted Dynamic Tensile Forming 

**Figure 21:**
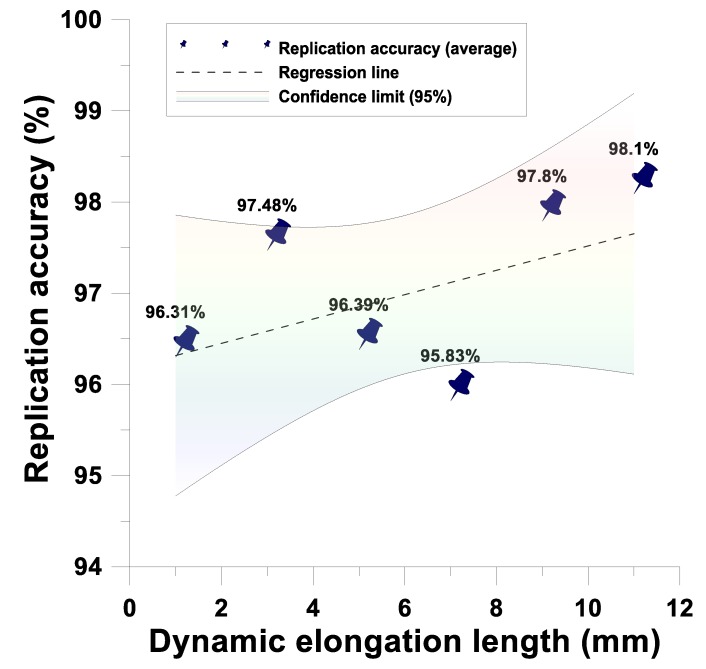
Dynamic tensile and gasbag-assisted imprinting process microstructure height imprint transcription rate.

**Figure 22:**
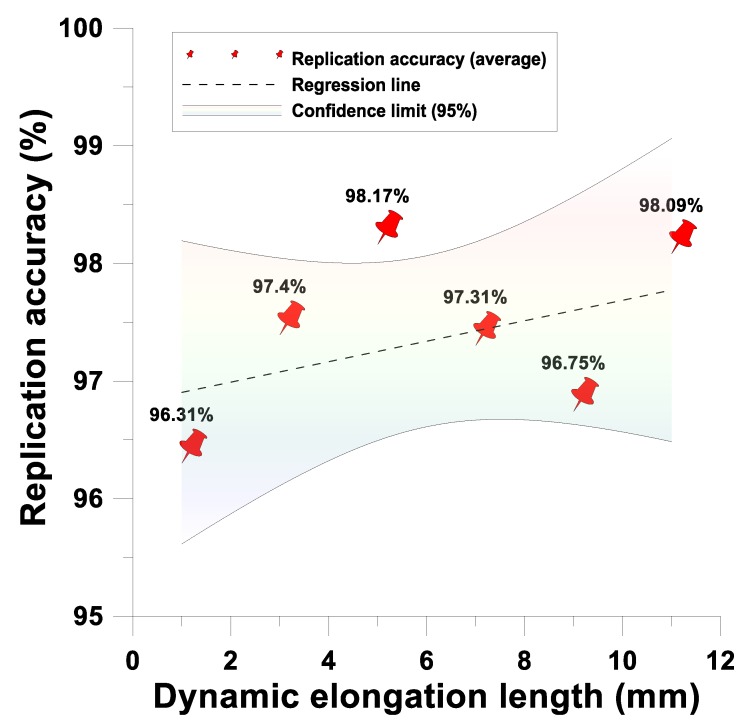
Dynamic tensile and gasbag-assisted imprinting process microstructure diameter imprint transcription rate.

The proposed system has simultaneous upper and lower cavities, thus, the dynamic tensile and gasbag-assisted imprinting process can be performed simultaneously. Therefore, the simultaneous forming process of the bifacial microstructure can be performed. For the array circular microstructure diameter of 180 μm, microstructure height of 130 μm feature dimensions, the dynamic tensile of 1 mm, 3 mm, 5 mm, 7 mm, 9 mm, and 11 mm in various directions were regulated. The gasbag-assisted imprint was measured by a surface profiler. The result showed that the imprint transcription rate of this system has accurate transfer printing height (overall average transcription rate: 96.985%) and transfer printing diameter (overall average transcription rate: 97.34%), and the transfer printing formability 95% confidence interval is established, as shown in Figure 21 and Figure 22. 

#### 4.3.3. Forming Operation Window of Bifacial Gasbag-Assisted Dynamic Tensile Imprint Reproduction 

**Figure 23:**
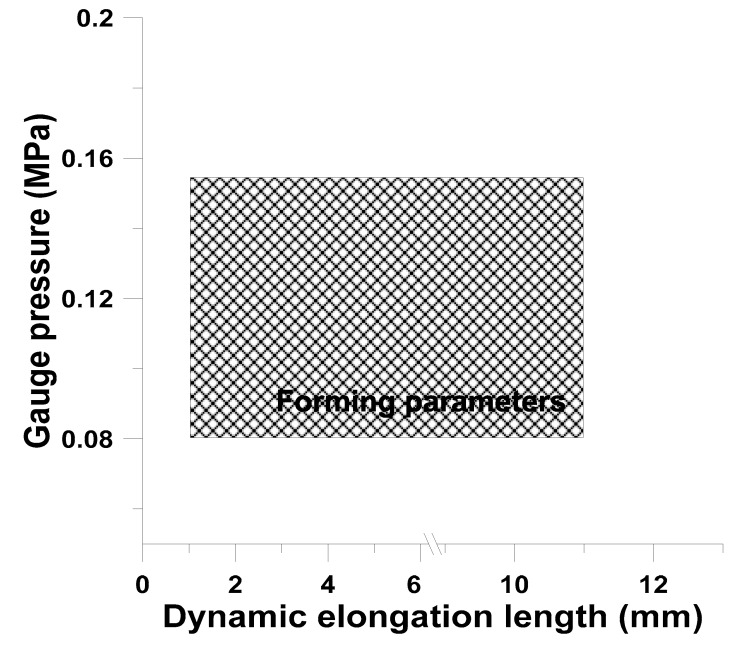
Dynamic tensile gasbag-assisted imprinting process reproduction forming operation window.

Regarding the array circular microstructure of the cross microstructure mold in the upper and lower cavities (diameter of 180 m, microstructure height of 130 m feature dimension), the dynamic tensile was performed to adjust 1 mm, 3 mm, 5 mm, 7 mm, 9 mm, and 11 mm in various directions. The gasbag-assisted imprinting process reproduction forming operation window was established by multiple experiments. The experimental results showed that, under the axial operating conditions, the gas-assisted pressure within 0.08Mpa~0.15Mpa has good reproduction formability, as shown in Figure 23. 

## 5. Conclusions

This study proposed a dynamic microstructure forming method for flexible polymer, and discussed the time-dependent creep behavior of dependent variables in the dynamic tensile process. Maxwell, Kelvin–Voight, Model A, and Model B were built for the cross microstructure mold. The findings showed that Model A has the steadiest performance. In terms of mold imprint uniformity, the flexible polymer mold imprint uniformity, mold pressure (stress, strain), and mechanical stress (von Mises stress and Tresca stress) effects in the tensile process were analyzed by Abaqus and ANSYS. The results showed that the system is uniform and stable. In terms of curved imprinting, for unequal curved imprinting, the geometric feature variation of the microstructure was calculated by MATLAB software. The results showed that the transverse deformation and longitudinal deformation are related to the location of the mold. In terms of asymmetric imprinting, an array cylinder with a diameter of 250um and a height of 75um was implemented. Different extensions were adjusted to find the similarities and differences in the forming of adjacent microstructures under curved imprinting. The single rectangular microstructure (1 × 1), array rectangular microstructure (4 × 4), array cylindrical microstructure, and array microlens hemispherical structure were calculated by asymmetric imprinting (MATLAB), as well as unequal rotation angle and tilt angle forming features, which are compared with the experiment to verify the feasibility of the prediction method. The result of imprint reproduction at rotation angle π/6° and tilt angle π/36° is close to the calculation result of the MATLAB software. 

## Figures and Tables

**Table 1:** Material parameters for Abaqus software simulation after testing the mechanical properties of the material.

Mechanical Properties	Gasbag	PDMS	Imprinting Platform
Density (Ton/mm^3^)	0.9	0.95	1.18
Young’s modulus (MPa)	35	2.46	3240
Poisson ratio	0.45	0.47	0.33

**Table 2:** Material properties test data under different (Polydimethylsiloxane, PDMS) mixing ratios (Agent A: Agent B) and test conditions.

Proportion	Loading Rate (mm/min)	Tension Stress (MPa)		Modulus (MPa)		Poisson’s Ratio		Shear Strength (N/cm^2^)
A:B								
	8	0.213		3.13		0.58		332.45
5:1	5	0.203		3.04		0.52		330.21
	2	0.197		2.92		0.53		298.31
	8	0.163		2.37		0.52		175.27
10:1	5	0.157		2.32		0.51		172.38
	2	0.158		2.28		0.49		166.97
	8	0.103		1.78		0.47		56.23
15:1	5	0.093		1.65		0.45		54.12
	2	0.071		1.60		0.48		50.92
Proportion	Loading rate (mm/min)		Strain rate (dε/dt) × 10^−4^		Strain (×10^−4^)		Stress relaxation (dS/dt) × 10^−4^	
A:B								
	8		38.095		337.328		89.519	
5:1	5		23.809		333.881			
	2		9.523		340.255			
	8		38.095		343.881		63.538	
10:1	5		23.809		338.362			
	2		9.523		346.491			
	8		38.095		289.325		6.711	
15:1	5		23.809		281.818			
	2		9.523		221.875			

**Table 3:** η value Mechanical mold derived from the four mechanical models.

Proportion A:B	Model (η Value)			
	Maxwell	Kelvin–Voight	Model A	Model B
5:1	1265.363631	−764.623189	2444.59607	1898.045447
10:1	1581.055442	−612.2271563	3284.394812	2371.583163
15:1	462.0473758	−59.29427477	1056.217947	693.0710637

**Table 4:** Contour features after uniaxial tensile curved imprinting forming.

Elongation Distance (mm)	Short Diameter (μm)	Long Diameter (μm)	Microstructures Height (μm)	Adjacent Height (μm)	Height Difference (μm)
0	230	230	67.75	70.12	2.36
2	228	243	64.63	68.19	3.55
4	228	252	63.27	67.25	3.97
6	225	255	61.15	65.16	4.01
8	225	257	58.04	63.13	5.09
10	223	262	56.89	62.80	5.90
